# Microarray analysis of breast cancer gene expression profiling in response to 2-deoxyglucose, metformin, and glucose starvation

**DOI:** 10.1186/s12935-022-02542-w

**Published:** 2022-03-19

**Authors:** Rita Aoun, Christopher El Hadi, Roula Tahtouh, Rita El Habre, George Hilal

**Affiliations:** 1 Cancer and Metabolism Laboratory, Faculty of Medicine, Saint-Joseph University, Beirut, Lebanon; 2 Faculty of Medicine, Saint-Joseph University, Beirut, Lebanon

**Keywords:** Breast cancer, Triple-negative breast cancer, Hormone receptor-positive subtype, 2-deoxyglucose, Metformin, Glucose starvation, Microarray

## Abstract

**Background:**

Breast cancer (BC) is the most frequently diagnosed cancer in women. Altering glucose metabolism and its effects on cancer progression and treatment resistance is an emerging interest in BC research. For instance, combining chemotherapy with glucose-lowering drugs (2-deoxyglucose (2-DG), metformin (MET)) or glucose starvation (GS) has shown better outcomes than with chemotherapy alone. However, the genes and molecular mechanisms that govern the action of these glucose deprivation conditions have not been fully elucidated. Here, we investigated the differentially expressed genes in MCF-7 and MDA-MB-231 BC cell lines upon treatment with glucose-lowering drugs (2-DG, MET) and GS using microarray analysis to study the difference in biological functions between the glucose challenges and their effect on the vulnerability of BC cells.

**Methods:**

MDA-MB-231 and MCF-7 cells were treated with 20 mM MET or 4 mM 2-DG for 48 h. GS was performed by gradually decreasing the glucose concentration in the culture medium to 0 g/L, in which the cells remained with fetal bovine serum for one week. Expression profiling was carried out using Affymetrix Human Clariom S microarrays. Differentially expressed genes were obtained from the Transcriptome Analysis Console and enriched using DAVID and R packages.

**Results:**

Our results showed that MDA-MB-231 cells were more responsive to glucose deprivation than MCF-7 cells. Endoplasmic reticulum stress response and cell cycle inhibition were detected after all three glucose deprivations in MDA-MB-231 cells and only under the metformin and GS conditions in MCF-7 cells. Induction of apoptosis and inhibition of DNA replication were observed with all three treatments in MDA-MB-231 cells and metformin-treated MCF-7 cells. Upregulation of cellular response to reactive oxygen species and inhibition of DNA repair mechanisms resulted after metformin and GS administration in MDA-MB-231 cell lines and metformin-treated MCF-7 cells. Autophagy was induced after 2-DG treatment in MDA-MB-231 cells and after metformin in MCF-7 cells. Finally, inhibition of DNA methylation were observed only with GS in MDA-MB-231 cells.

**Conclusion:**

The procedure used to process cancer cells and analyze their expression data distinguishes our study from others. GS had the greatest effect on breast cancer cells compared to 2-DG and MET. Combining MET and GS could restrain both cell lines, making them more vulnerable to conventional chemotherapy.

**Supplementary Information:**

The online version contains supplementary material available at 10.1186/s12935-022-02542-w.

## Background

Breast cancer (BC) is the most common female malignancy and the second leading cause of cancer-related death in women [1], with more than 500,000 deaths worldwide each year [2]. Based on molecular marker expression, BC is divided into subtypes as follows: hormone receptor (HR)-positive subtype (estrogen receptor (ER +) or progesterone receptor (PR +)), human epidermal growth factor receptor 2 overexpression subtype (HER2 +), and triple-negative breast cancer (TNBC) (ER − , PR −, HER2 −) [3]. The subtypes of TNBC present as basal-like 1 (BL1), basal-like 2 (BL2), immunomodulatory (IM), mesenchymal (M), mesenchymal stem-like (MSL), and luminal androgen receptor (LAR) [4]. This classification into subtypes is useful when selecting the most appropriate therapy.

For patients with HR-positive BC, endocrine therapy is the preferred treatment approach. The most common adjuvants are the estrogen modulator Tamoxifen and the aromatase inhibitors (letrozole, anastrozole, and exemestane), while chemotherapy is reserved for patients at risk of visceral crisis or with endocrine resistance [5]. For patients with HER2-positive BC, the combination of an anti-HER2 monoclonal antibody (Trastuzumab, Pertuzumab) with chemotherapy is the standard treatment [6]. Compared with other types of breast cancer, TNBC is the most difficult subtype to treat because it lacks appropriate targets for molecular therapy; therefore, chemotherapy remains the main approach to first-line treatment of TNBC [7]. However, some cases of BC develop resistance to chemotherapies or fail to respond to conventional therapies due to altered tumor glucose metabolism [8]. This aberrant metabolism is characterized by hyperactivated aerobic glycolysis, a phenomenon coined “Warburg effect” [9], and decreased oxidative phosphorylation (OXPHOS). Controlling these phenomena using metabolic inhibitors such as the antidiabetic agent metformin (MET) and 2-deoxyglucose (2-DG), or by inducing glucose starvation (GS) should therefore be a promising approach to overcome therapy resistance [10].

The combination of chemotherapeutic drugs and MET is being clinically tested for efficacy in the treatment of cancer. The credibility of MET use is related to MET's ability to target cancer cell metabolism and interrupt tumor progression [11]. MET-mediated inhibition of mitochondrial respiratory chain complex 1 increases levels of adenosine monophosphate (AMP), which activates AMP-activated protein kinase (AMPK). The latter activation in turn inhibits the mammalian target of Rapamycin (mTOR), which is responsible for cell proliferation [12]. In addition, AMPK activation suppresses many metabolic processes (gluconeogenesis, protein and fat synthesis, glycolysis, and fatty acid beta-oxidation) [13]. It also induces cell cycle arrest and apoptosis through down-regulation of the tumor protein p53 (p53) and exerts anti-inflammatory actions by inhibiting nuclear factor kappa B (NF-κB) pathways [14].

The anticancer agent 2-DG is also considered to target the metabolic homeostasis of a cell. Clinical trials have suggested that combining 2-DG with chemotherapies overcomes drug resistance by eliminating breast cancer stem cells (BCSC)-induced resistance [15]. This glucose decoy is phosphorylated by hexokinase 2 (HK2) to inhibit glycolysis, resulting in adenosine triphosphate (ATP) depletion and inducing BC cell death [16]. 2-DG can alter the redox state of a cell, leading to apoptosis. It can also contribute to the inhibition of protein glycosylation, leading to endoplasmic reticulum (ER) stress and activation of the unfolded protein response (UPR) [17].

Glucose addiction in cancer is linked to hyperproliferation [18]. Inhibition of glycolytic activity via GS could be a potential therapeutic approach [19]. Clinical studies have shown a correlation between caloric intake and breast cancer etiology. In addition, a lower incidence of cancer and a longer life span in men are observed after a low-sugar diet. GS inhibits energy-dependent signaling pathways, including the insulin-like growth factor-1/phosphoinositide 3-kinase/protein kinase B/mTOR (IGF-1/PI3K/Akt/mTOR) pathway, and activates AMPK resulting in down-regulation of glycolysis activity and cell proliferation [20]. In addition, GS increases ketone bodies in the bloodstream, which protects mitochondria from inflammation and reactive oxygen species (ROS) during cancer treatment [21]. It also decreases lactate production to prevent intracellular alkalinization [22].

Several microarray studies have analyzed the transcriptomic response of cancers, specifically breast cancer, to different modalities of targeting glucose metabolism. Publications experimenting with anti-glycolytic approaches (including 2-DG, MET, and GS) have shown upregulation [23–26] or downregulation [27–36] in recurrent pathways and functions, including cell cycle; DNA replication, damage, repair; chromosome organization; nuclear division; and antigen processing and presentation via Major histocompatibility complex class I (MHC class I).

BC cells, among other cancers, are known to depend mainly on glucose to proliferate. This phenomenon is being exploited to develop new cancer treatments which may include glucose-lowering drugs and/or a low glucose diet in their protocols. In this study, we decided to investigate the difference in biological functions between glucose-lowering drugs (2-DG, metformin) and glucose starvation (GS) by analyzing differentially expressed genes (DEGs) in estrogen and progesterone receptor positive (MCF-7) and triple-negative (MDA-MB-231) BC cell lines using microarray analysis. What distinguishes our study from others is the procedure used to process cancer cells and analyze their gene expression data. All reviewed cell culture studies using glucose challenge as an anticancer approach starved cancer cells of glucose for up to 48 h, which differs from our GS that lasted for two weeks. In addition, we performed two data mining protocols using different DEG identification and functional enrichment methods to obtain more robust results, and we identified common up-and down-regulated functions between cell lines and between treatments within each cell line.

## Methods

### Reagents

2-Deoxy-D-glucose (2-DG), 1,1-dimethyl biguanide hydrochloride (Metformin), penicillin/ streptomycin solution (100 × ), fetal bovine serum (FBS), trypsin solution, Dulbecco's modified Eagle’s medium (DMEM) (Glucose-free, L-glutamine, phenol red, sodium pyruvate and sodium bicarbonate powder suitable for cell culture), Dulbecco’s modified Eagle's medium high glucose (DMEM, 4500 mg/L), Dulbecco's modified Eagle’s medium low glucose (DMEM, 1000 mg/L) were bought and imported from Sigma-Aldrich (St. Louis, MO, USA). NucleoZol was purchased from MACHEREY–NAGEL (Bethlehem, PA, USA). GeneChip™ WT PLUS reagent kit was obtained from Thermo Fisher Scientific (Waltham, MA, USA).

### Cell culture

BC cell lines MDA-MB-231 (ATCC^®^HTB-26™) and MCF-7 (ATCC^®^HTB-22™) were purchased from the American type culture collection (ATCC, Manassas, Virginia, USA). Cells were grown in DMEM 4500 mg/L glucose supplemented with 10% FBS and 1% penicillin/streptomycin (100 × ) and treated with 20 mM MET or 4 mM 2-DG for 48 h. GS was executed by gradually decreasing the glucose concentration in the culture medium from 4.5, to 1, to 0.5 g/L, until a concentration of 0 g/L was reached. All culture media were supplemented with 10% FBS and 1% penicillin/streptomycin (100 × ). Cells already maintained in medium containing 4.5 g/L glucose were transferred to another medium containing 1 g/L glucose for 48 h and then maintained in 0.5 g/L glucose for five days. After that, the cells were kept in a zero-glucose medium for one week, with 10% FBS containing about 4.0 mmol/L of glucose, which is the average glycemia in fasting individuals that mimics physiology, and 1% penicillin/streptomycin 100x. The culture medium was changed daily. Cells were maintained at 37 °C in a humidified atmosphere, with 5% CO2.

### RNA extraction and gene expression microarrays

Total RNA from three independent experiments (biological replicates) was extracted using NucleoZol according to the manufacturer's instructions. RNA concentration and A260/A280 ratio were determined using the NanoDropTM 1000 spectrophotometer (Thermo Scientific). RNA integrity was assessed by denaturing agarose gel electrophoresis (1%). One hundred ng of total RNA was reverse transcribed following the instructions of the GeneChip^®^ WT Plus Reagent Kit (Affymetrix, Inc., Santa Clara, CA, USA). Complementary RNA (cRNA) was synthesized and amplified through in vitro transcription (IVT) using T7 RNA polymerase (using the Affymetrix WT cDNA Synthesis and Amplification Kit). The transcription was done having second-stranded cDNA as a template. After purification, the sense strand cDNA was synthesized by reverse transcription of the cRNA using 2nd cycle primers. The cRNA template was then hydrolyzed using RNase H., and 5.5 µg of ss-cDNA purified, fragmented, and labeled with biotin according to the Affymetrix WT End Labeling Kit. The ss-cDNA was then hybridized to the Clariom™ S human transcriptome array (Affymetrix, Inc., Santa Clara, CA, USA) at 45 °C for 17 h using the GeneChip™ 645 hybridization oven. The arrays were washed, stained on the FS450 Fluid Station, and scanned using the GeneChip Scanner 3000 7G (Affymetrix, Inc., Santa Clara, CA, USA) according to the GeneChip™ User Guide. The raw CEL files containing the intensity data were extracted analyzed as described in the following.

## Statistical and bioinformatics analysis

### Gene analysis protocols

Two microarray data mining protocols were adopted, each using a different method for finding the differentially expressed genes and for clustering functional terms. The findings from the two methods were then unified, which should add statistical certainty to our results. The gene expression data were extracted in each protocol and CEL files were generated using the Thermo Fisher Transcriptome Analysis Console (TAC) with the SST-RMA as the normalization algorithm [37]. Each strategy has its limitations but when combined they added valuable data checks to the final results.

**Fig. 1:**
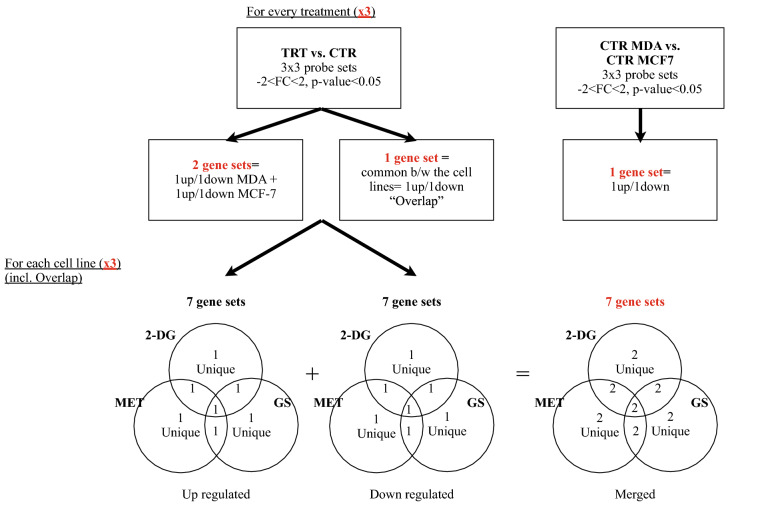
A simplified schematization of the partitioning of DEGs

Both protocols start by comparing, in TAC, three sets of treatment probes to three sets of control probes in each cancer cell line separately. Genes with a "Fold Change" (FC) <  − 2 or > 2 and having a p-value < 0.05 were regarded as significantly differentially expressed. The interrogated genes were then divided into two groups: those that are upregulated (i.e., FC > 2) and those that are downregulated (i.e., FC < − 2). The "Volcano plots" for each comparison were exported simultaneously. In addition, the overlapping genes between the two cancer types in each of the upregulated and downregulated treatments, and the set merging the latter two, were determined using "BioinfoGP Venny" [38]. This latter set will be referred to as the "overlap" set. The intersection of the sets of up-regulated and then down-regulated genes of the three treatments in each cell line, including the overlap, was also performed using Venny. Then, each subsection of the intersected Venn diagram was merged with its other pair. Another final set was queried from the comparison of controls in each of the two lines, still using the same FC criteria. A simplified schematic of the DEGs’ partitioning is shown in Fig. 1.

### First protocol

The first protocol proceeded by composing the gene sets in the Database for Annotation, Visualization, and Integrated Discovery (DAVID) for gene enrichment analysis and clustering [39]. This method measures functional relationships between genes or terms by the kappa coefficient. Selected enrichment terms included the gene ontology (GO) GOTERM_ Biological Process (BP)_DIRECT, GOTERM_ Cellular Component (CC) _DIRECT, and GOTERM_ Molecular Function (MF) _DIRECT and the Kyoto Encyclopedia of Genes and Genomes (KEGG) and Reactome for pathway enrichment. The DAVID functional annotation clustering tool was used, and clusters were exported to Excel files for further analysis. A significant number of non-clustered terms were relatable to the clustered terms. The latter had a p-value < 0.05 and a Benjamini p-value rapidly becoming insignificant with lower cluster "Enrichment Scores". To account for the precedent, a geometric mean < 0.2 of the p-value of all terms of a cluster was considered sufficient to retain this cluster. On the other hand, if no significant cluster was found, the non-clustered terms with a Benjamini p-value < 0.05 were retained, and/or the analysis was checked with the results of the second protocol.

### Second protocol

For the second protocol, the programming language for statistical computing "R" was used [40]. Comparison of treatments to controls within each cancer cell line, and between cell controls, was performed using significance analysis of microarray (SAM) [41]. The data type was set to "two-class unpaired" and the FC option "log scale" was checked. The "fitness factor ∆" was retained when it corresponded to a 90th percentile FDR < 0.005, a median FDR as small as possible, and "called genes" as high as possible (> 100 genes). The number of permutations was adjusted from a default value of 100 to 5000 when the cited requirements were not met. As a result, the ∆ and overall FDR values varied faintly around 0.5 and 0.002, respectively, and all gene FCs happened to be either < − 2 or > 2. The results were then exported with their "SAM plots" and the up-and down-regulated sets were extracted. Intersections were performed using packages that are part of R, "gplots" and "ggVennDiagram" [42, 43], and the same sets were drawn, including those of the "overlap" cell line. GO and Reactome pathway enrichment analyses were then performed on the sets, and terms with a Benjamini p-value < 0.05 were retained only. The R packages "AnnotationDbi", "clusterProfiler" and "ReactomePA" were required for gene enrichment [44–46]. The terms were then converted into three semantic similarity matrices and 1 gene overlap similarity matrix, namely three for GOs and one for Reactome respectively. These matrices were subjected to "binary cut" clustering using "simplifyEnrichment" [47], as recommended by the software authors. The results were ordered and exported for analysis. It should also be noted that binary cut is not recommended for KEGG terms, which were covered by the first protocol.

### Cluster analysis

In each protocol, clusters in the merged set (i.e., containing both up-and down-regulated genes) were labeled "up-/down-regulated" based on their cluster matches in the analyzed up-or down-regulated sets. It should be noted that the analysis of the subsets resulting from the intersection of the three treatments was performed using only BP GO and Reactome, and only common genes between treatments were analyzed. The previous decisions were made to avoid redundancy. Finally, the results from both methods were unified and presented as-is in this paper. The BP GO and Reactome clusters provided by the second protocol, using binary clustering, best summarizes our results and so they were visualized using diagrams showing the statistical significance of the clusters, outlined by the most relevant term. Bar plots for the precedent data and others showing DEGs were constructed using "cowplot," "ggplot2," and "dplyr" [48–50]. Apple's "Pages" application was useful for representing intersections in the Venn diagrams, which are not to scale. BP GO heat maps for the main comparisons (i.e. treatments vs. controls) were constructed from the data provided by the second method using "simplifyEnrichment" with binary clustering as the default method.

## Results

**Fig. 2:**
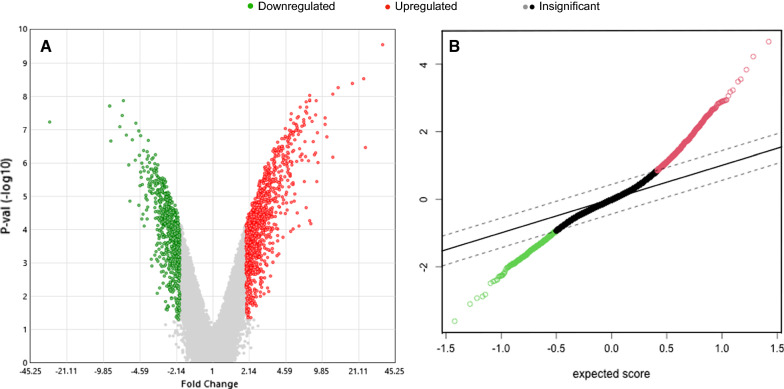
Microarray-based expression profiling. Volcano plot (on the left) and SAM plot (on the right) representation of the DEGs in a comparison of MDA-MB-231 cell line subjected to GS vs. non-treated cells. **a** The plot shows − log10 (p-value) (y-axis) and log2 (fold change) (x-axis). The significant cut-off was set to a p-value of 0.05 (− log10 (p-value) ≥  − 2, horizontal line), the biological cut-off was set to a fold change of ± twofold (log2 (fold change) <  − 1 and >  + 1, vertical lines). **b** The observed score is SAM’s d_i_ score of every gene before the permutation process. The expected score is the score calculated after permutation. The pointed lines are the Δ thresholds we ought to choose

The analysis was performed on two BC cell lines, MDA-MB-231 and MCF-7. DEGs were identified between the controls of the two cell lines, the controls and treatments, and between treated cells, (i.e., 2-DG, MET, or glucose-starved cells). DEGs were determined using TAC or SAM. DEGs were visualized either on a volcano or SAM plot (Fig. 2a, b) (only the representation of MDA-MB-231-GS versus the MDA-MB-231 control is shown). The lists of DEGs and the others volcano and SAM plot representations are shown in Additional file 1: Table S1. Biological significance was extracted from the gene lists by systematically enriching their DEGs with GO and pathway terms (KEGG and Reactome for DAVID, only Reactome for binary clustering). The top enriched clusters are only described in this section. The comparison between the cell line’s controls can be reviewed with Additional file 2 and Additional file 3: Figure S1.

### MDA-MB-231 cell line

#### Regulation with 2-DG

**Fig. 3:**
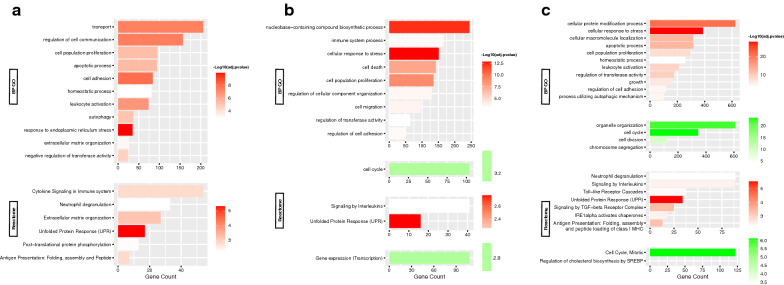
Functional enrichment analysis of DEGs in MDA-MB-231 cell line. Summary plot of the BP GO and Reactome terms representing each functional cluster in MDA-MB-231 cell line: **a** 2-DG- treated cells, **b** MET**-**treated cells, and **c** cells subjected to GS vs. non-treated cells. The most relevant upregulated (red) and downregulated (green) functions in each experiment are shown, sorted by –log10(adjusted p-value), and ranked by the terms’ corresponding number genes. All terms are of adjusted p-value < 0.01

For the MDA-MB-231 cell line, the comparison of 2-DG-treated cells with the control (Fig. 3a) revealed that most clusters are up-regulated and very few are down-regulated. For the upregulated biological processes: response to stimulus (lipids, hormones); response to ER stress, and UPR (chaperone activation); cellular component localization (protein, vesicle-mediated); cell migration; immune system processes (antigen presentation, neutrophil degranulation, and toll-like receptors (TLRs) interleukin (IL) pathway signaling); cell adhesion (laminin and proteoglycans); autophagy; protein phosphorylation and catabolism by ubiquitination; apoptotic processes; cell population proliferation; temperature homeostasis; and organization of extracellular components (collagen and hemidesmosome). These processes were found to recruit molecular functions describing protein binding, enzymes (kinase, ubiquitin ligase), adhesion molecules (cadherin), and glutamate receptors. In addition, protein disulfide isomerase, mitogen-activated protein kinase (MAPK), and transmembrane transporter activities were observed. Cellular components included cytoplasmic vesicles and lysosomes, nucleus and ER network, ER lumen and chaperone complex, cell adhesion foci, and the extracellular region.

Down-regulated clusters show DNA replication, cell cycle (G1/S phase transition), and purine/pyrimidine metabolism.

#### Regulation with metformin

Also comparing MDA-MB-231 cells, DEGs in MET-treated cells versus control (Fig. 3b) yielded mostly up-regulated clusters with a few down-regulated clusters. For the up-regulated clusters, the biological processes were RNA biosynthesis and protein phosphorylation; stress and stimulus response (ROS, UPR, hormones) and associated signal transduction (cyclic adenosine monophosphate (cAMP); catalytic activity (ubiquitin-protein transferase/ligase); apoptosis; cell migration; antigen processing and presentation via MHC class I; and transcription mediated by the forkhead box O (FOXO) transcription factor. Molecular functions were primarily binding mechanisms of “sequence-specific” transcriptional DNA cis-regulatory regions, proteins such as transcription (co-)factors or repressors, identical proteins (homo-dimerization), and kinases. Cellular components of activity included the extracellular exosome, focal adhesion, and cell-substrate junctions.

For the down-regulated clusters, the biological processes were DNA replication; DNA damage response and repair (resolution of D-loop structures, homologous DNA repair (HDR) by homologous recombination (HRR), Double-strand breaks (DSBs)) and cell cycle (cyclin-dependent G1/S phase transition); chromatin organization.

#### Regulation during glucose starvation

Finally, for glucose-starved cells (Fig. 3c), clustering also yielded mostly up-regulated with slightly more down-regulated clusters. For the up-regulated clusters, the biological functions were protein metabolism and modification process (post-translational modification (PTM), phosphorylation, proteolysis) and macromolecule biosynthesis; RNA transcription by RNA polymerase II; response to stress and stimulus (ER-related, UPR and chaperones); apoptosis; antigen processing and presentation via MHC class I; temperature homeostasis; cell adhesion; and transforming growth factor-beta (TGFβ) receptor signaling. Molecular functions were mainly binding and homodimerization of "domain-specific" proteins (c-terminal) and binding of enzymes (protein kinase, GTPase and ubiquitin(-like) ligase), anions, misfolded proteins, ribonucleoprotein complexes, and cadherins. Cellular components include the cytoplasm (organelle membranes, mitochondria, vesicles and lysosomes, ER and its chaperone complex, Golgi apparatus), protein complexes (transferase, ubiquitin ligase), focal adhesions, and anchoring junctions.

For the down-regulated clusters, biological processes showed DNA methylation and replication; cell cycle (polo-like kinase 1 (PLK1) activity during the G2/M transition); chromatin and microtubule organization and nuclear fission; DNA damage response (Ataxia telangiectasia-mutated (ATM) and ataxia telangiectasia and Rad3-related (ATR) upregulation in response to replication stress); DNA repair (DSBs processing); protein and RNA cellular localization and chromosome segregation; NOTCH signaling; cholesterol biosynthesis (CB) via sterol regulatory element protein (SREBP) gene expression; FOXO-mediated transcription.

### MCF-7 cell line

#### Regulation with 2-DG

**Fig. 4:**
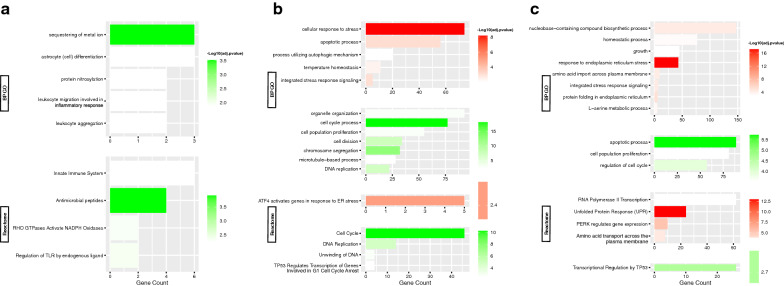
Functional enrichment analysis of DEGs in MCF-7 cell line. Summary plot of the BP GO and Reactome terms representing each functional cluster in MCF-7 cell line: **a** 2-DG- treated cells, or **b** MET**-**treated cells, or **c** cells subjected to GS vs. non-treated cells. The most relevant upregulated (red) and downregulated (green) functions in each experiment are shown, sorted by –log10(adjusted p-value), and ranked by the terms’ corresponding number genes. All terms are of adjusted p-value < 0.01

As with the MCF-7 cell line, comparison of the 2-DG-treated cells with the control (Fig. 4a) yielded only down-regulated clusters, in contrast to what the same comparison yielded for the MDA-MB-231 cell line. The biological processes were zinc ion homeostasis, cell differentiation, innate immune response, and Rho GTPase-activated nicotinamide adenine dinucleotide phosphate (NADPH) oxidases activity.

#### Regulation with metformin

Comparison of MET versus control (Fig. 4b) yielded mostly down-regulated and a few up-regulated clusters. For the up-regulated clusters, the biological properties were: cellular responses to stress (ROS, and hormone); UPR (protein kinase R (PKR)-like ER kinase (PERK)-mediated); ER stress (transcription factor 4 (ATF4)-mediated gene activation) and signal transduction by the class mediator p53; cellular autophagy; apoptosis; and temperature homeostasis. No molecular function terms were shown due to the paucity of DEGs. Vesicles, lytic vacuoles, and cytoplasm were sites of activity.

For the down-regulated clusters, the biological functions were: cell cycle (G1/S phase transition and associated P53 regulation); DNA damage response; chromosome segregation; DNA replication (DNA unwinding); RNA transcription via RNA polymerase II promoter; nuclear division, chromosome and cytoskeleton organization; cell population proliferation.

#### Regulation during glucose starvation

To end, glucose starvation versus control (Fig. 4c), resulted in predominantly up-regulated clusters and some down-regulated clusters. For the up-regulated clusters, the biological processes were cell response to ER stress (ATF4 activation), UPR (Activating transcription factor 6-alpha (ATF6α) and X-box 1 (S) binding protein (XBP1[S]) activate chaperone genes, PERK-mediated response, inositol-requesting enzyme 1 α (IRE1 α)-mediated response); cell growth; transcription via RNA polymerase II; protein phosphorylation; serine family amino acid biosynthetic process; transmembrane transport of amino acids and hexoses (mediated by ABC family proteins) and cellular localization; metals and cations homeostatic process; and FOXO mediated transcription. Molecular functions include transmembrane transport of amino acids and anions, binding of misfolded proteins, kinases and ubiquitin (-like) ligases, homodimerization of proteins, and DNA binding of cofactors and transcription repressors. Cellular components include the cytoplasm, the ER chaperone complex, and kinase complexes (cyclin-dependent holoenzyme, serine/threonine).

As for the down-regulated clusters, their biological functions were as follows: apoptotic process; mitotic cell cycle (G1/S phase transition and associated transcriptional regulation by P53); and cell population proliferation.

### Overlapping cell line

To show the functional similarities between the two BC cell types, a gene overlap sham cell line was established. Overlapping up-and down-regulated genes between the two cell lines were identified for each treatment. They were analyzed and cross-referenced in the same manner as the true cell lines. It should be mentioned that the overlapping DEGs in the 2-DG treatment revealed too few genes, allowing no enrichment.

#### Regulation with metformin

**Fig. 5:**
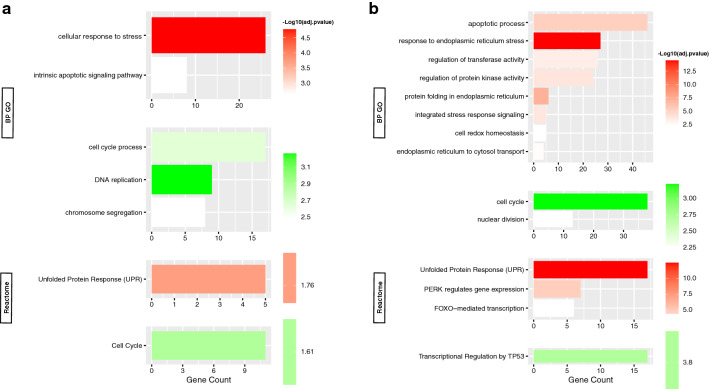
Functional enrichment analysis of DEGs in “overlap” cell line. Summary plot of the BP GO and Reactome terms representing each functional cluster in “overlap” cell line: **a** MET-treated cells or **b** cells subjected to GS vs. non-treated cells. The most relevant upregulated (red) and downregulated (green) functions in each experiment are shown, sorted by  −  log10(adjusted p-value), and ranked by the terms’ corresponding number genes. All terms are of adjusted p-value < 0.01

Enrichment with MET resulted primarily in up-regulated clusters and some down-regulated clusters (Fig. 5a). For the up-regulated clusters, the biological processes were a cellular response to UPR, ER stress (intrinsic apoptotic signaling), and other stimuli (glucocorticoids, hormones, lipids, chemicals); and RNA biosynthesis and metabolic process. Molecular functions describe the binding of “sequence-specific” DSBs to DNA (transcription regulatory region), and transcription factors. Cellular components include the nucleus, chromosome, RNA polymerase II transcription regulator, and ubiquitin ligase protein complexes.

For the down-regulated clusters, the biological processes were DNA replication; and cell cycle (chromosome segregation, cyclin E-associated events during the G1/S transition, Skp1-Cullin-1-F-box (SCF)-mediated degradation of p27/p21, S-phase kinase-associated protein (Skp2)).

#### Regulation during glucose starvation

DEGs in the GS primarily resulted in up-regulated clusters (Fig. 5b). For these, the biological processes were as follows: ER stress response (ATF4-mediated gene activation) and UPR; protein folding in the ER (chaperone activation via ATF6α); apoptosis; protein phosphorylation (cyclin-dependent kinase and MAPK activity); RNA polymerase II biosynthesis in response to stress; transferase activity; cell localization (ER to cytosol transport, chromosome); cell motility and migration; and FOXO-mediated transcription. Molecular functions describe the binding of misfolded proteins, carbohydrate derivatives, and anions; intramolecular disulfide isomerase activity (S–S bond transposition); chaperone-mediated protein folding; and transmembrane transport of neutral amino acids. Cellular components include the cytoplasm, granule (melanosome), ER smooth membrane, chaperone complex, ER quality control compartment, and cell ruffle and leading edge.

For the down-regulated clusters, the biological processes were cell cycle arrest (p53 regulation of transcription of genes involved in G2 cell cycle arrest, PLK-mediated events, cyclin-associated events); chromosome segregation; response to DNA damage; and nuclear fission and organelle organization.

BP GO heat maps of the previous conditions are shown in Additional file 4: Figure S2, Additional file 5: Figure S3, and Additional file 6: Figure S4.

### Intersection of DEGs

**Fig. 6:**
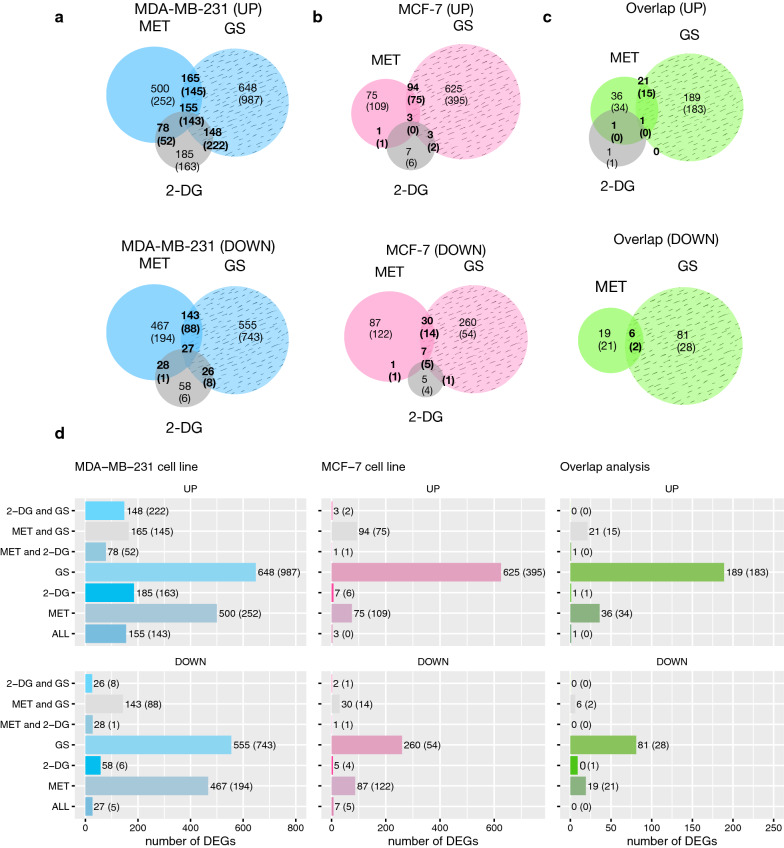
The intersection of DEGs in MDA-MB-231, MCF-7, and “overlap” cell lines following the treatment with 2-DG, MET, and GS. Venn diagrams displaying the number of upregulated and downregulated DEGs determined using TAC and SAM in **a** MDA-MB-231, **b** MCF-7, and **c** “overlap” cell lines. **d** Bar plots presenting the difference in DEGs of the cell lines. Results from SAM are shown in parenthesis. Statistical criteria: p-value < 0.05, FC <  − 2 or > 2

For this part, genes from the three treatments were intersected and Venn diagrams constructed. These give the number of intersected DEGs in the MDA-MB-231 (Fig. 6a) and MCF-7 (Fig. 6b), and overlap cell lines (Fig. 6c). The gene lists are present in Additional file 7: Table S2, Additional file 8: Table S3, and Additional file 9: Table S4).

#### Intersection in the MDA-MB-231 cell line

Starting with the MDA-MB-231 cell line (Fig. 6a), genes common between MET and 2-DG treatments were analyzed and their enrichment didn’t show any statistically significant clusters. Enrichment of the intersected genes of 2-DG and GS treatments yielded only up-regulated clusters. We cite cellular response to ER stress and UPR; protein localization, and vesicle-mediated transport; cell migration; HER2 and TGFβ tyrosine kinase signaling; cell differentiation; cell adhesion; autophagy; phosphorus metabolism and protein PTM; apoptosis.

Genes resulting from the intersection of MET and GS yielded predominantly up-regulated clusters with functions such as response to lipopolysaccharides (LPS) and lipids; tumor necrosis factor (TNF) signaling pathway, NOD-like receptor (NLR) signaling pathway; apoptosis; cell proliferation; cell differentiation; and cell–cell adhesion. For down-regulated clusters, we have a response to DNA damage stimuli; DNA replication; DNA repair; protein phosphorylation, and ubiquitination; mitotic cell cycle; and chromosome organization.

Finally, the genes shared by all treatments mainly exhibited up-regulated clusters with biological functions such as response to lipids and LPS, cytokines, and ER stress and UPR; transferase activity; kinase activity; RNA transcription by RNA polymerase II; ROS production; endogenous peptide antigen presentation by MHC class I; cell population proliferation; cell component movement and cell motility; apoptosis. As for the down-regulated clusters, we have the cell cycle and DNA repair.

#### Intersection in the MCF-7 cell line

Moving to the MCF-7 cell line (Fig. 6b), few or no genes were found in common between the MET and 2-DG comparison, and thus no significant enrichment resulted. Comparison of 2-DG and GS treatments yielded only upregulated clusters, namely neurotrophic tropomyosin receptor kinase (NTRKs) signaling.

Common DEGs between MET and GS treatments yield a predominantly up-regulated enrichment cluster and a down-regulated cluster. For the upregulated, biological functions showed cellular response to oxygen-containing compounds, UPR and ER stress, and starvation (saccharides, lipids, peptides); apoptosis, signaling via the class mediator p53; cell proliferation; temperature homeostasis; metabolic processes of amino acids; regulation of transcription from the RNA polymerase II promoter in response to stress; transmembrane transport of amino acids; and autophagy. The down-regulated cluster exhibited cell cycle processes (microtubule-binding).

Genes common to all treatments showed only clusters of down-regulation. The cellular processes involved were protein nitrosylation; lipid response; cellular zinc ion homeostasis; cell development; intrinsic apoptotic signaling pathway, and activity of the cysteine-type endopeptidase involved in the apoptotic process (NFκB activity); Rho GTPase effectors.

#### Intersection in the overlap cell line

The intersection of treatment genes was only possible between MET and GS (Fig. 6c). One up-regulated cluster resulted from enrichment, having biological functions including cellular response to ER stress and UPR; and signaling through apoptotic signaling pathways.

Additional bar plots (Fig. 6d) also show the difference in the number of DEGs between the overlap and the two cell lines.

## Discussion

We compared the effect of glucose deprivation on MDA-MB-231 and MCF-7 BC cell lines using microarray data mining methods to establish a database for DEGs and pathways modulations. Two data extraction methods were adopted, each with complementary advantages to the other.

Our results showed that MDA-MB-231 cells exposed to any of the three treatments resulted in ER stress response, activation of the UPR pathway, apoptosis, and inhibition of cell cycle and DNA replication, which goes in line with past research on the matter [51, 52]. For MCF-7, ER stress and subsequent UPR were also observed with MET and GS. MET also downregulated DNA damage response, RNA biosynthesis and transcription, and cell population proliferation. Accumulation of misfolded proteins is known to induce ER stress and activate an ER-specific adaptive response, the UPR. Intracellular protein aggregation due to slow protein folding is observed under conditions such as lack of nutrients (e.g. glucose) as well as oxygen deprivation, oxidative stress (OS) [53], abnormalities of calcium ion homeostasis, and protein glycosylation [54, 55]. The UPR process aims to restore intracellular homeostasis by inducing adaptation pathways related to the activation of three ER transmembrane proteins: IRE1, PERK, ATF4, and ATF6.

We also demonstrated that MET and GS treatment of MDA-MB-231 cells leads to ROS production and inhibition of DNA repair. GS and high concentrations of MET (10–40 mM, as seen in our study) are known to sensitize the highly glycolytic metastatic TNBC cell line (i.e. MDA-MB-231) to apoptosis through increased mitochondrial ROS and mitochondrial membrane potential (MMP) disruption [56–59]. MET-treated MCF-7 cells showed increased cellular stress, response to oxygen-containing compounds, and apoptosis. MET treatment is known to interfere with signaling pathways related to OS and cell survival, as treatment increases nuclear p53 expression [60], and AMPK phosphorylation [61, 62]. MET-induced reduction in viability mediated by apoptosis has also been demonstrated in our results and studies on the subject [63–65]. This apoptosis in BC-derived cells could be caused by ROS-independent mitochondrial dysfunction [63], which was also shown to specifically target cancer cells with no effect on normal breast epithelial cells [65].

Our results further indicate that all three treatments downregulate DNA replication in MDA-MB-231 through suppression of nucleotide metabolism [66]. During the cell cycle, deregulation of DNA replication leads to chromosomal alterations, promoting tumorigenesis [67]. In our study and others [68], all treatments downregulated the G1/S phase transition in the MDA-MB-231 cell line, with GS also affecting PLK1 activity during the G2/M transition. For the MCF-7 cell line, MET triggered the inhibition of cell proliferation and associated RNA biosynthesis as well as checking DNA damage, specifically at the p53-regulated G1/S transition. Such proliferation arrest has been described by the majority of MET studies with MCF-7 cells [61, 69, 70]. With the one-week glucose-starved MCF-7 cells, apoptosis was deregulated as well as the cell cycle and cell proliferation. This deregulation is echoed by other papers describing the same process [71–73]. That being said, GS has also been shown to increase invasiveness and metastasis of MCF-7 tumor cells. This phenomenon might be explained by stimulation of urokinase plasminogen activator (uPA) and plasmin activity [74].

We also showed that MET downregulates DNA repair mechanisms in MDA-MB-231 cells. Compared to normal cells, malignant cells have a higher DNA damage response (DDR), aimed at maintaining genome integrity. They stimulate DNA repair capacity to cope with DNA damage and to survive. MET downregulates RAD51 by ubiquitination, a key player in homologous recombination DNA repair, leading to inhibition of the DNA damage repair pathway [75]. Furthermore, ATM and ATR signaling cascades are two key pathways that initiate DDR and are activated in response to DSBs [76]. We found that GS downregulated ATR activation in response to replication stress, leading to downregulation of DNA repair mechanisms. Therapies inhibiting ATR are currently being tested in early-phase clinical trials in advanced solid tumors [77].

Our results indicated that 2-DG treatment increases collagen, laminin, and proteoglycan organization. Collagen serves as a scaffold for the ECM and its excessive production is an indicator of BC malignancy [78, 79]. In addition, proteoglycans are associated with high invasiveness and progression of BC, and the adhesive protein laminin plays an important role in the development of BC [80]. The role of adhesion molecules is to maintain cell-to-cell contact and attachment to the extracellular matrix. Loss of cell adhesion has classically been considered a pro-tumorigenic feature, promoting metastasis and invasion of tumor cells [81]. Adhesion molecules such as E-cadherin, can exhibit a decreased expression in BC due to methylation of its promoter in TNBC [82].

The results also revealed that MET in the MDA-MB-231 cell line increases calcium signaling and activates cAMP signaling. Calcium-based mechanisms have been shown to be crucial for the induction of apoptosis [83], and the role of cAMP as a growth suppressor reduces the metastatic properties of TNBC [84]. Besides, MET enhances the expression of human leukocyte antigen (HLA)-encoded MHC I in the MDA-MB-231 cell line [85]. This is advantageous considering that HLA downregulation is frequently observed in tumors and is thought to correlate with disease progression [86]. In addition, it is well established that MHC I expression on tumor membranes is essential for tumor destruction by cytotoxic T lymphocytes (CTLs). Additionally, expression of interferon (IFN)-associated molecules in BC also depends on MHC I molecules, with being associated with a good response to anthracycline-based chemotherapy [87].

Our results thus suggested that MCF-7 cells were less responsive to the glucose challenges than the MDA-MB-231 cell line. The latter conclusion was drawn from the fact that fewer modulated genes, less significance and number of enrichment terms, and fewer biological functions related to cell lethality and dysfunction were observed with the MCF-7 cell line. The difference in the cell lines' expression of hormone receptors and the resulting difference in growth factor-associated pathways should explain, to some degree, the difference in the cell lines' behaviors to the same treatments. Adopting the same conclusion strategy, we can also say that starving cells for glucose had the most significant impact on both cell lines, compared with MET and 2-DG treatments. Cells exposed to MET were slightly less responsive than those undergoing GS, but significantly more reactive than cells exposed to 2-DG. The latter assumptions originate from the fact that GS was more enriched and associated with terms of functions related to cell lethality and dysfunction, with MET coming in second place for drug responsiveness.

Owing to the limitations of any in vitro study, the results of our in vitro study must be confirmed under in vivo conditions. In addition, further studies should determine the effect of the duration of exposure to the three glucose challenges on cancer cells, which should also be performed on a larger number of cells in order to generalize from specific cell lines to the subtypes that these lines represent. Finally, it would be interesting to see studies on the response of cancer cells to a combination of glucose challenges, particularly GS and MET, seeing their rich impact on cancer cells and the different processes they regulate.

## Conclusion

The ultimate goal of this study was to investigate the procedure that can replicate in the most beneficial way possible the effect of glucose-lowering on the vulnerability of BC cells. Our study showed that the three different glucose deprivations modes had remarkable effects on both BC cell lines. 2-DG appears to be the “gentlest” on the cells’ genes modulation. GS for one week in the presence of FBS had the greatest influence on the cells. It should be noted that the MDA-MB-231 cell line responded better than the MCF-7 cell line to all three treatments.

Our results suggest that the combination of MET and GS may be a beneficial approach to inhibit both MDA-MB-231 and MCF-7 cell lines, which are triple-negative and HR-positive breast cancer cell lines respectively. By stressing the cancer cells, they decrease their proliferation and enter an energy-saving mode, making the cancer cells more vulnerable and likely more sensitive to conventional treatments at lower doses, all while avoiding most of the side effects on normal cells. Further studies should be performed on cell lines representing these two BC subtypes to generalize from specific cell lines to subtypes representing these lines. Finally, these in vitro observations should be validated using an in vivo model in combination with drugs for the treatment of breast cancer.

## Supplementary Information

**Additional file 1: Table S1.** Data analysis of the microarray experiments. Lists of DEGs in MDA-MB-231 and MCF-7 cell lines determined by TAC or SAM with volcano and SAM plots representations (fold changes of ± twofold; p-value < 0.05).

**Additional file 2:** Comparison of controls.

**Additional file 3: Figure S1.** Functional enrichment analysis of DEGs in MDA-MB-231 and MCF-7 cell lines. Heat maps of the BP GO terms in the upregulated clusters in **a** MDA-MB-231 and **b** MCF-7 controls cell lines.

**Additional file 4: Figure S2.** Functional enrichment analysis of DEGs in MDA-MB-231 cell line. Heat maps of the BP GO terms in MDA-MB-231 cell line: **a** 2-DG- treated cells, **b** MET-treated cells, and **c** cells subjected to GS vs. non-treated cells. The GO enrichment terms are used to calculate a matrix defining the similarity between every two terms. Binary clustering is then performed, revealing non-overlapping clusters (the red mesh) of similar terms. Redundant keywords for each cluster are shown on the right.

**Additional file 5: Figure S3.** Functional enrichment analysis of DEGs in MCF-7 cell line. Heat maps of the BP GO terms in MCF-7 cell line: **a** 2-DG- treated cells, or **b** MET-treated cells, or **c** cells subjected to GS vs. non-treated cells. The GO enrichment terms are used to calculate a matrix defining the similarity between every two terms. Binary clustering is then performed, revealing non-overlapping clusters (the red mesh) of similar terms. Redundant keywords for each cluster are shown on the right.

**Additional file 6: Figure S4.** Functional enrichment analysis of DEGs in “overlap” cell line. Heat maps of the BP GO terms in “overlap” cell line: **a** MET-treated cells or **b** cells subjected to GS vs. non-treated cells. The GO enrichment terms are used to calculate a matrix defining the similarity between every two terms. Binary clustering is then performed, revealing non-overlapping clusters (the red mesh) of similar terms. Redundant keywords for each cluster are shown on the right.

**Additional file 7: Table S2.** Lists of intersected DEGs in MDA-MB-231 cell line determined by TAC or SAM and displayed in Venn diagram (fold changes of ± twofold; p-value < 0.05).

**Additional file 8: Table S3.** Lists of intersected DEGs in MCF-7 cell line determined by TAC or SAM and displayed in Venn diagram (fold changes of ± twofold; p-value < 0.05).

**Additional file 9: Table S4.** Lists of intersected DEGs in “overlap” cell line determined by TAC or SAM and displayed in Venn diagram (fold changes of ± twofold; p-value < 0.05).

## Data Availability

The datasets used and/or analyzed during the current study are available from the corresponding author on reasonable request.

## Abbreviations

2-DG: 2-Deoxyglucose
ATCC: American type culture collection
ATF4: Activating transcription factor 4
ATF6-α: Activating transcription factor 6-alpha
AMP: Adenosine monophosphate
ATP: Adenosine triphosphate
AMPK: AMP-activated protein kinase
ATM: Ataxia telangiectasia-mutated
ATR: Ataxia-telangiectasia and Rad3-related
BL1: Basal-like 1
BL2: Basal-like 2
BCSCs: Breast cancer stem cells
BC: Breast cancer
BP: Biological process
CC: Cellular Component
CB: Cholesterol biosynthesis
cRNA: Complementary RNA
cAMP: Cyclic adenosine monophosphate
CTLs: Cytotoxic T lymphocytes
DAVID: Database for annotation, visualization, and integrated discovery
DEG: Differentially expressed genes
DMEM: Dulbecco’s Modified Eagle’s Medium
DSBs: Double strand breaks
PERK: Endoplasmic reticulum protein kinase R-like kinase
ER: Endoplasmic reticulum
IRE1 α: Inositol requiring enzyme 1 α
ER +: Estrogen receptor
ECM: Extracellular matrix
Skp2: F-box 2 protein associated with the S-phase kinase
FC: Fold change
FBS: Fetal Bovine Serum
FOXO: Forkhead box O transcription factor
GO: Gene Ontology
GS: Glucose starvation
HK2: Hexokinase 2
HDR: Homologous DNA repair
HRR: Homologous recombination
HR: Hormone receptor
HER2: Human epidermal growth factor receptor 2
HLA: Human leukocyte antigen
IM: Immunomodulators
IGF-1: Insulin-like growth factor 1
IFN: Interferon
IL: Interleukin
IVT: in vitro Transcription
KEGG: Kyoto Encyclopedia of Genes and Genomes
LPS: Lipopolysaccharides
LAR: Luminal androgen receptor
MHC class I: Major histocompatibility complex class I
mTOR: Mammalian target of rapamycin
MSL: Mesenchymal stem-like
M: Mesenchymal
MET: Metformin
MMP: Mitochondrial membrane potential
MAPK: Mitogen-activated protein kinase
MF: Molecular Function
NTRKs: Neurotrophic tropomyosin receptor kinases
NADPH: Nicotinamide adenine dinucleotide phosphate oxidase
NLR: NOD type receptor
BNFκB: Nuclear factor-kappa
OXPHOS: Oxidative phosphorylation
OS: Oxidative stress
PI3K: Phosphoinositide 3-kinase
PLK1: Polo-like kinase 1
PTM: Post-translational modification
PR +: Progesterone receptor
AKT: Protein kinase B
PKR: Protein kinase R
ROS: Reactive oxygen species
DDR: Response to DNA damage
UPR: Response to unfolded proteins
SAM: Significance Analysis of Microarrays
SCF: Skp1-Cullin-1-F-box
SREBP: Sterol regulatory element binding protein gene
TLR: Toll-like receptor
TAC: Transcriptome Analysis Console
TGFβ: Transforming growth factor beta
TNBC: Triple-negative breast cancer
TNF: Tumor necrosis factor
p53: Tumor protein p53
uPA: Urokinase plasminogen activator
XBP1(S): X-box 1(S) binding protein
