# Supplementary material for: Microarray analysis of breast cancer gene expression profiling in response to 2-deoxyglucose, metformin, and glucose starvation
Source: Cancer Cell Int. 2022 Mar 19;22:123. doi: 10.1186/s12935-022-02542-w (PMC8933915; doi:10.1186/s12935-022-02542-w)
Supplement: Supplementary file 2 — Additional file 2: Comparison of controls. [file 12935_2022_2542_MOESM2_ESM.pdf]

Comparison of controls

a) Upregulation in the MDA-MB-231 cell line

For clusters up-regulated in the MDA-MB-231 control, and inversely down-regulated in the MCF-7 control, the biological functions were the upregulation of [positive regulation of] the extracellular matrix of the cytoskeleton (actin, myosin, microtubule assembly), organelles (nucleus and endomembrane system), cell junction, and cell projection organization; [negative regulation of] cell growth (via growth factors); [positive regulation of] cell motility (amoeboid movement) and vesicular-mediated transport to the membrane (secretion, degranulation); [positive regulation of] cell differentiation and organ development; [positive regulation of] signaling (Rho GTPase, HER2), response to abiotic chemicals, amino and retinoic acids, metal ions (sequestration of copper, zinc, cadmium), lipids and lipopolysaccharides (LPS), hypoxia, and chemotaxis; metabolic processes of phosphorus, ribonucleotides, lipids (phospholipids and triglycerides), and glycosaminoglycans (GAGs); [positive regulation of] protein (self-) phosphorylation (c-Jun Nterminal kinase (JNK), mitogen-activated protein kinase [MAPK]), aminoacylation of cytosolic transfer ribonucleic acid [tRNA]); body fluid and zinc homeostasis; cell death and apoptosis; and [positive regulation of] cell proliferation. Molecular functions describe catalytic activities (hydrolase, transferase, GTPase, kinase, cyclic nucleotide phosphodiesterase) and protein binding; cytoskeleton (actin, vinculin, tubulin), collagen, adhesion molecules (cadherin, integrin), metal ions (via metallothionein), SH3 protein domain and purine nucleotide binding. Cellular compartments include the cytoplasm (cortical), cell-substrate junction, muscle cell cytoskeleton, cell leading edge, and projections (actin-based and neuronal-like, lamelli, filo, and invadopodium), secretory vesicles and granules (endomembrane system and extracellular exosome), and cell surface.

b) Upregulation in the MCF-7 cell line

As with the upregulated MCF-7 control clusters, thus downregulated in the MDA-MB-231 control, biological functions show upregulation of [negative regulation of] nucleic acid biosynthesis (RNA polymerase I promoter opening, RNA polymerase II transcribes small nuclear RNA [snRNA] genes to regulate transcription, RNA polymerase III from type I/II promoters), lipid/fatty acid metabolism (OXPHOS, biosynthesis), protein modification, and metabolism; autophagy; intracellular transport and protein localization (nucleocytoplasmic, post-Golgi, vesicular-mediated); cell signaling mediated by (small) GTPases (in response to DNA damage, induced senescence, hormones [Ras pathway]) ; organization of chromosomes (prophase condensation, telomere packaging) and mitochondria (respiratory complex); regulation of cell size; [negative regulation of the] mitotic cell cycle ; G2/M DNA damage checkpoint and repair (doublestrand break [DSB] end processing and recruitment of signaling proteins, ATM [Ataxia telangiectasia-mutated] phosphorylation, and non-homologous end joining [NHEJ]); cellular development and differentiation; mitochondrial translation processes; and xenobiotic metabolism. Molecular functions primarily describe protein binding, DNA/nucleic acid (double-stranded (ds), sequence-specific, transcription regulatory region), metal ions, transcription factors to DNA (repressor, RNA polymerase II specific), and (small) GTPases and kinases; oxidoreductase (acting on CH-OH, NAD[P]H, donor RNAs with NAD[P] or quinone as acceptors) and methyltransferase activities. Cellular compartments include cytoplasm, membrane organelles, nucleoplasm, chromatin, ribosome (organelle), and mitochondria; and catalytic protein complexes (transferase, endonuclease/ribonuclease, RNA polymerase III, vesicle attachment, respiratory chain).
