# Supplementary figures and images for: Microarray analysis of breast cancer gene expression profiling in response to 2-deoxyglucose, metformin, and glucose starvation
Source: Cancer Cell Int. 2022 Mar 19;22:123. doi: 10.1186/s12935-022-02542-w (PMC8933915; doi:10.1186/s12935-022-02542-w)

a

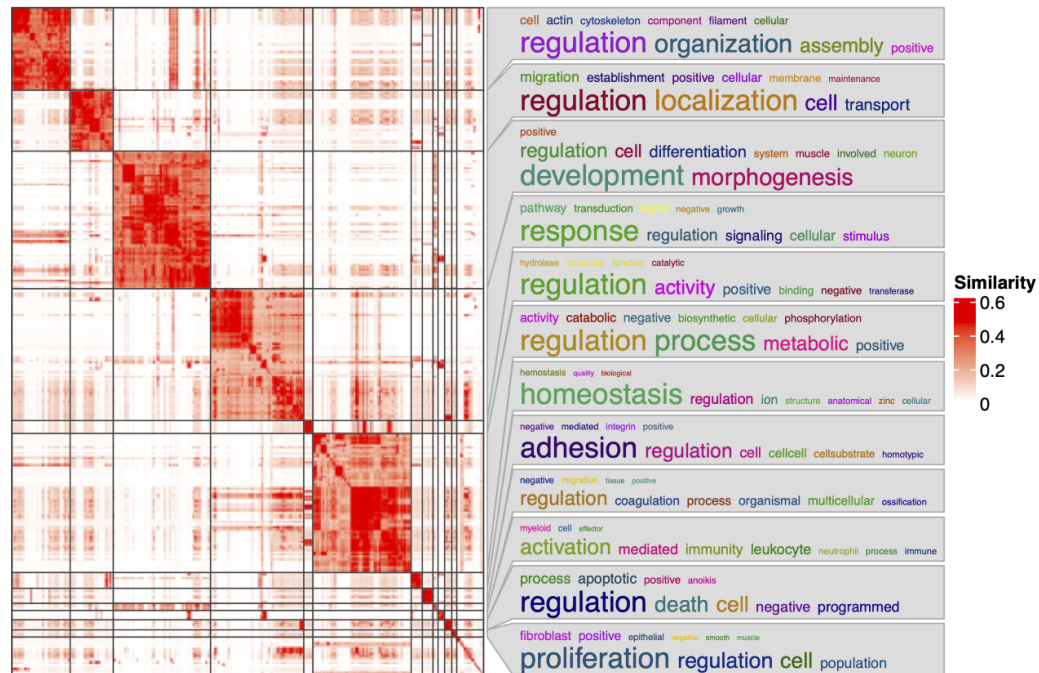

b

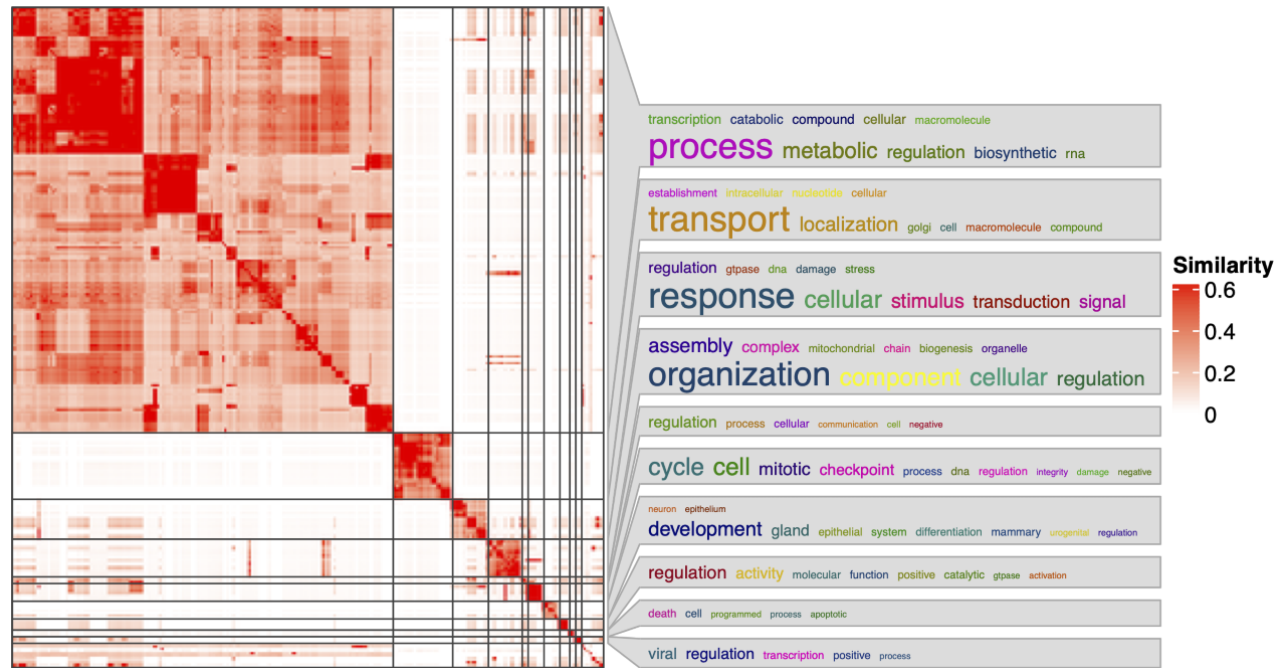

Supplement: Supplementary file 3 — Additional file 3: Figure S1. Functional enrichment analysis of DEGs in MDA-MB-231 and MCF-7 cell lines. Heat maps of the BP GO terms in the upregulated clusters in a MDA-MB-231 and b MCF-7 controls cell lines. [file 12935_2022_2542_MOESM3_ESM.pdf]

a

2-DG vs. CTR

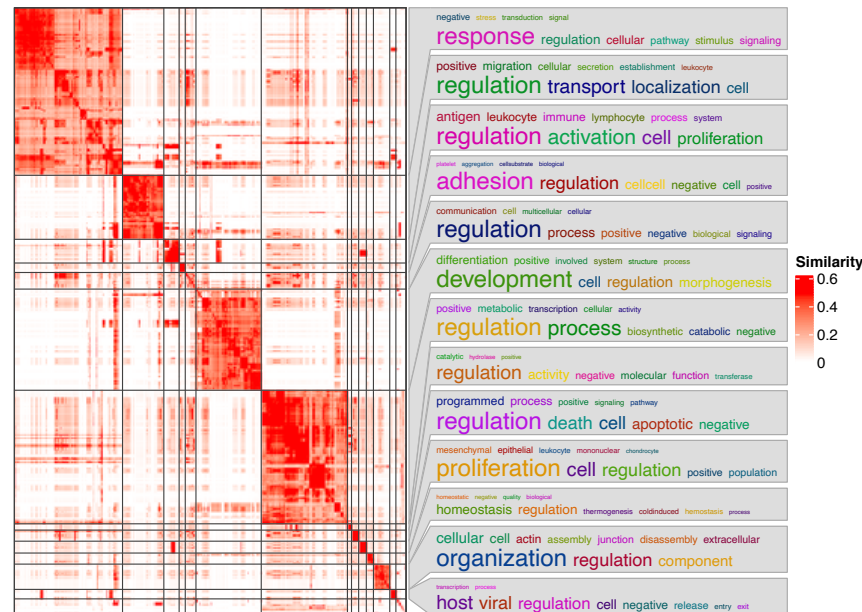

b

MET vs. CTR

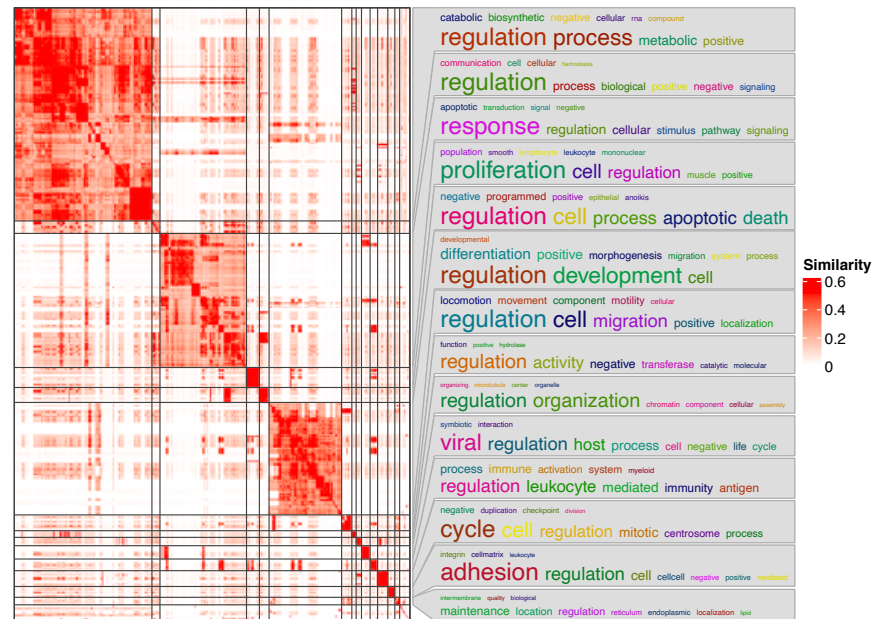

c

GS vs. CTR

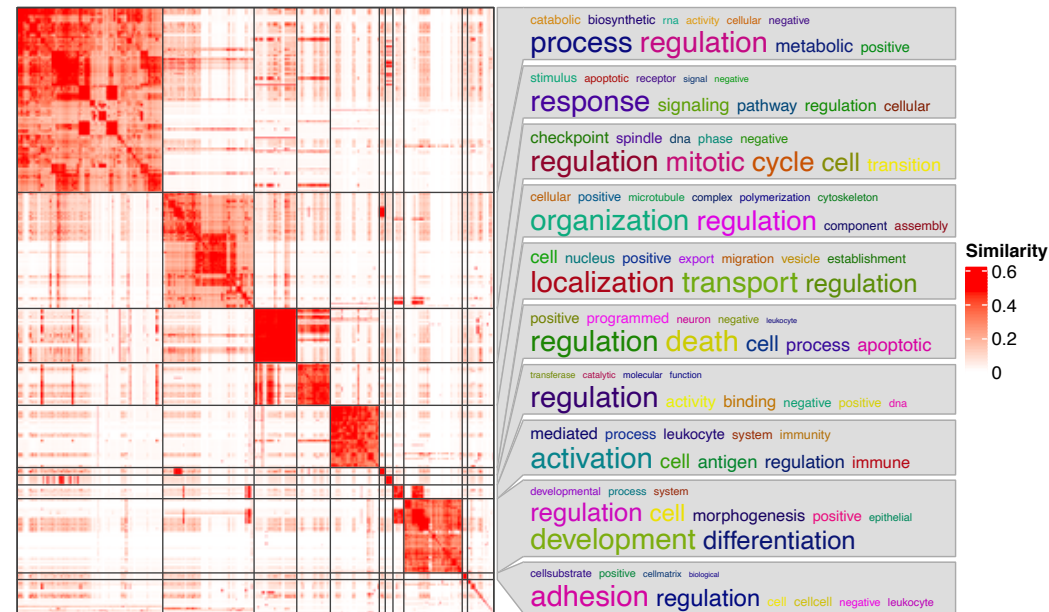

Supplement: Supplementary file 4 — Additional file 4: Figure S2. Functional enrichment analysis of DEGs in MDA-MB-231 cell line. Heat maps of the BP GO terms in MDA-MB-231 cell line: a 2-DG- treated cells, b MET-treated cells, and c cells subjected to GS vs. non-treated cells. The GO enrichment terms are used to calculate a matrix defining the similarity between every two terms. Binary clustering is then performed, revealing non-overlapping clusters (the red mesh) of similar terms. Redundant keywords for each cluster are shown on the right. [file 12935_2022_2542_MOESM4_ESM.pdf]

a

2-DG vs. CTR

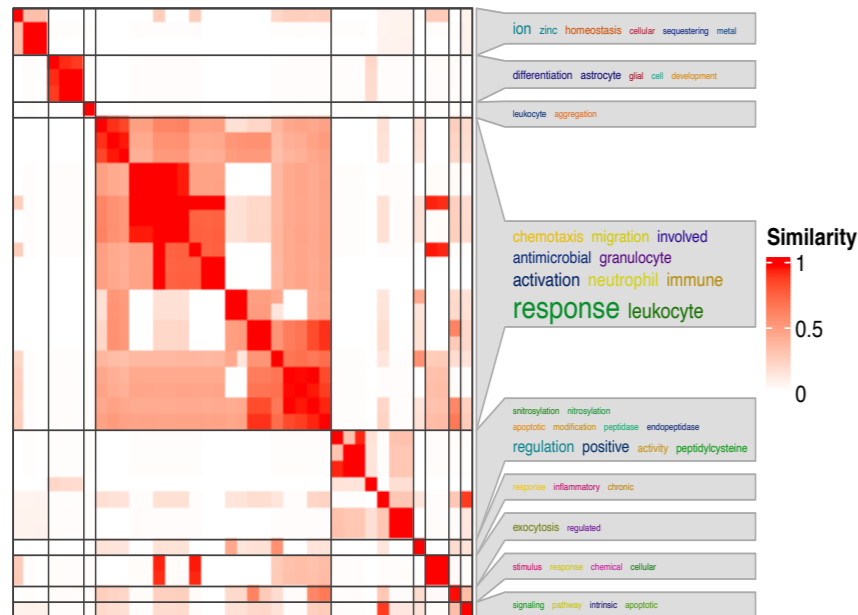

b

MET vs. CTR

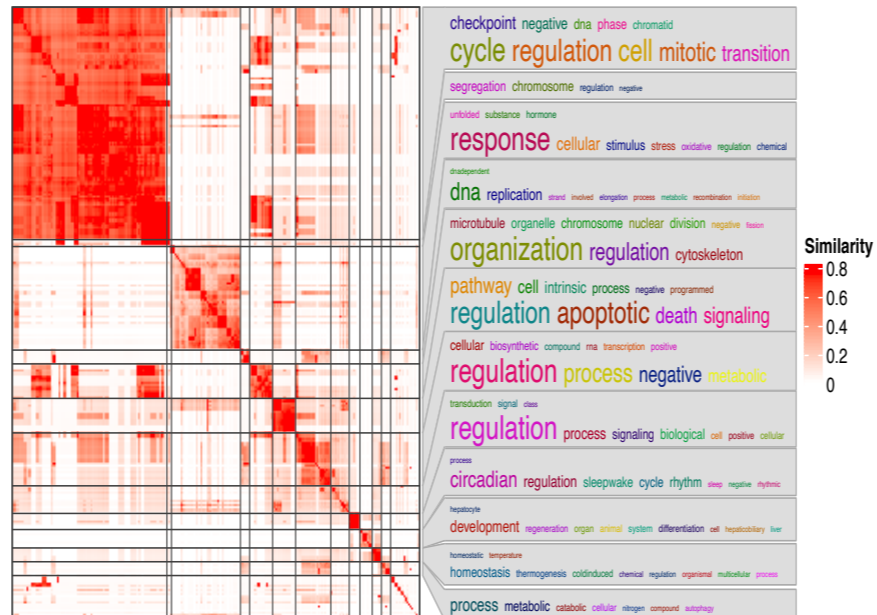

c

GS vs. CTR

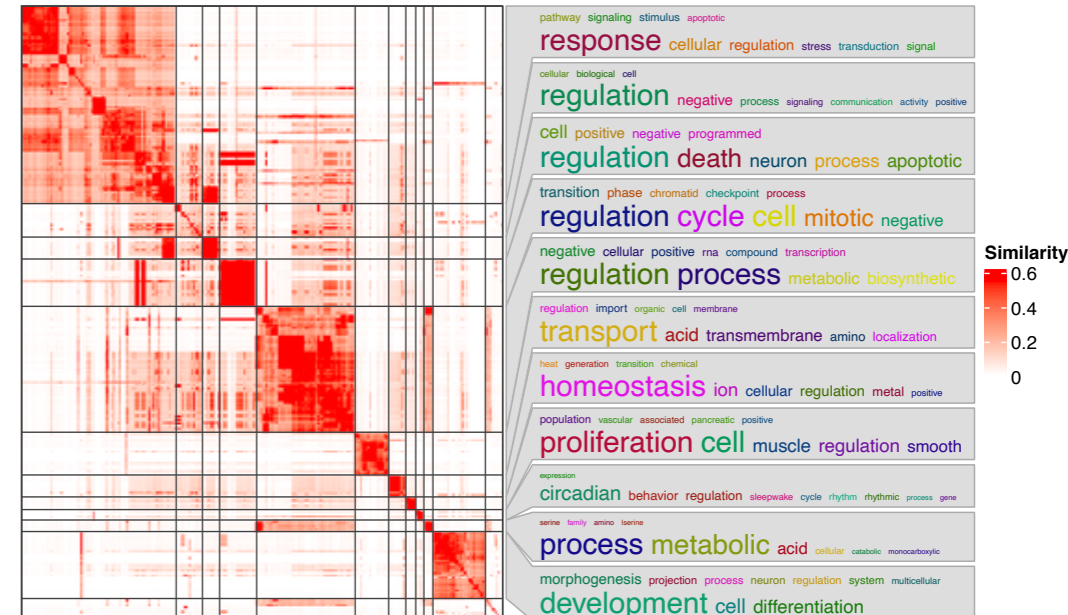

Supplement: Supplementary file 5 — Additional file 5: Figure S3. Functional enrichment analysis of DEGs in MCF-7 cell line. Heat maps of the BP GO terms in MCF-7 cell line: a 2-DG- treated cells, or b MET-treated cells, or c cells subjected to GS vs. non-treated cells. The GO enrichment terms are used to calculate a matrix defining the similarity between every two terms. Binary clustering is then performed, revealing non-overlapping clusters (the red mesh) of similar terms. Redundant keywords for each cluster are shown on the right. [file 12935_2022_2542_MOESM5_ESM.pdf]

a

MET vs. CTR

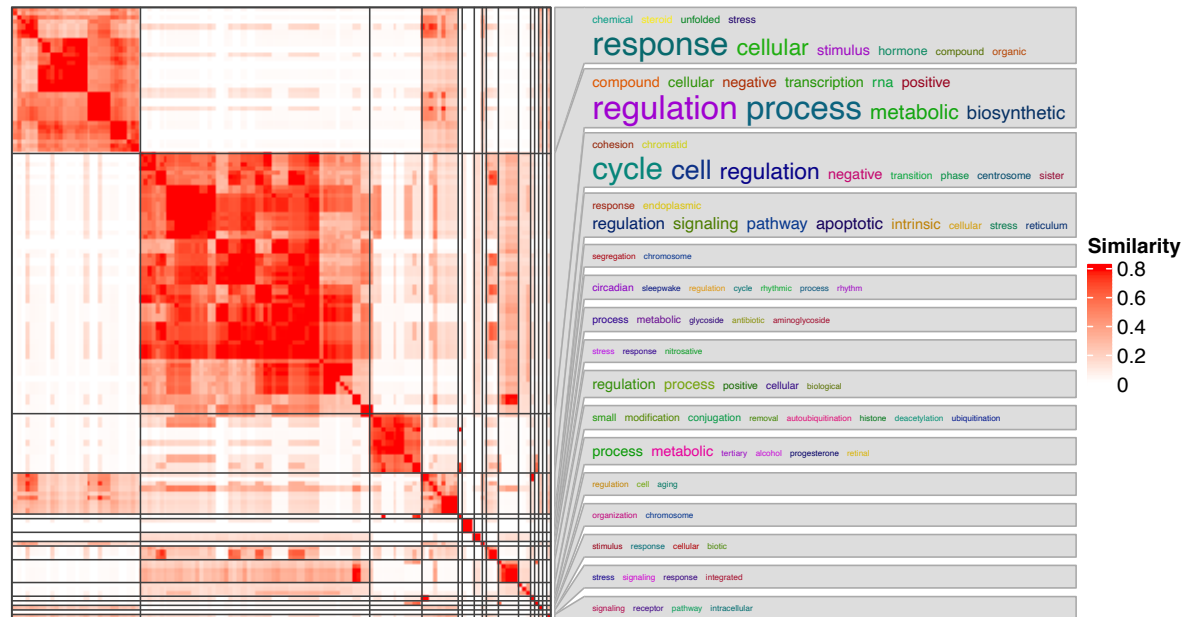

b

GS vs. CTR

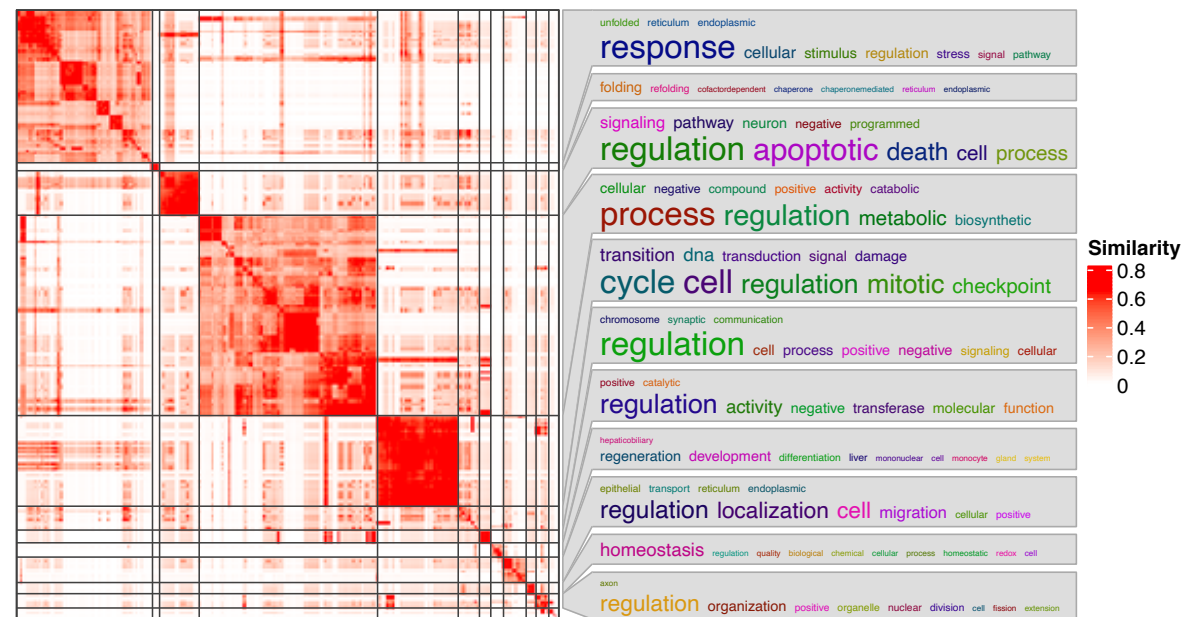

Supplement: Supplementary file 6 — Additional file 6: Figure S4. Functional enrichment analysis of DEGs in “overlap” cell line. Heat maps of the BP GO terms in “overlap” cell line: a MET-treated cells or b cells subjected to GS vs. non-treated cells. The GO enrichment terms are used to calculate a matrix defining the similarity between every two terms. Binary clustering is then performed, revealing non-overlapping clusters (the red mesh) of similar terms. Redundant keywords for each cluster are shown on the right. [file 12935_2022_2542_MOESM6_ESM.pdf]
